# The Influence of Two Different Invitation Letters on Chlamydia Testing Participation: Randomized Controlled Trial

**DOI:** 10.2196/jmir.2907

**Published:** 2014-01-30

**Authors:** Gill ten Hoor, Christian JPA Hoebe, Jan EAM van Bergen, Elfi EHG Brouwers, Robert AC Ruiter, Gerjo Kok

**Affiliations:** ^1^Maastricht UniversityDepartment of Work & Social PsychologyMaastrichtNetherlands; ^2^Public Health Service South LimburgDepartment of Sexual Health, Infectious Disease and Environmental HealthGeleenNetherlands; ^3^Maastricht UniversityDepartment of Medical MicrobiologyMaastrichtNetherlands; ^4^STI AIDS NetherlandsAmsterdamNetherlands; ^5^AMC - University of AmsterdamDepartment of General PracticeAmsterdamNetherlands

**Keywords:** invitation letter, chlamydia, screening, testing, behavior change theories

## Abstract

**Background:**

In the Netherlands, screening for chlamydia (the most prevalent sexually transmitted infection worldwide) is a relatively simple and free procedure. Via an invitation letter sent by the public health services (PHS), people are asked to visit a website to request a test kit. They can then do a chlamydia test at home, send it anonymously to a laboratory, and, within two weeks, they can review their test results online and be treated by their general practitioner or the PHS. Unfortunately, the participation rates are low and the process is believed to be not (cost-) effective.

**Objective:**

The objective of this study was to assess whether the low participation rate of screening for chlamydia at home, via an invitation letter asking to visit a website and request a test kit, could be improved by optimizing the invitation letter through systematically applied behavior change theories and evidence.

**Methods:**

The original letter and a revised letter were randomly sent out to 13,551 citizens, 16 to 29 years old, in a Dutch municipality. Using behavior change theories, the revised letter sought to increase motivation to conduct chlamydia screening tests. The revised letter was tailored to beliefs that were found in earlier studies: risk perception, advantages and disadvantages (attitude), moral norm, social influence, and response- and self-efficacy. Revisions to the new letter also sought to avoid possible unwanted resistance caused when people feel pressured, and included prompts to trigger the desired behavior.

**Results:**

No significant differences in test package requests were found between the two letters. There were also no differences between the original and revised letters in the rates of returned tests (11.80%, 581/4922 vs 11.07%, 549/4961) or positive test results (4.8%, 23/484 vs 4.1%, 19/460). It is evident that the new letter did not improve participation compared to the original letter.

**Conclusions:**

It is clear that the approach of inviting the target population through a letter does not lead to higher participation rates for chlamydia screening. Other approaches have to be developed and pilot tested.

## Introduction

In a 3-year systematic register-based yearly chlamydia screening project in three regions in the Netherlands, all 16 to 29 year old citizens were given the opportunity at no charge to test for Chlamydia trachomatis. Via an invitation letter sent by the public health services (PHS), they were asked to visit a website [1] where they could request a test kit. Subsequently, they could do a chlamydia test at home, send it anonymously to a laboratory, and, within two weeks, they would be able to review their test results online and could be treated by their general practitioner or the PHS. Despite this free and relatively simple procedure, receiving the letter triggered only a small number of young people to participate. In the first round in 2008, the participation rate was 16.1%. The rate decreased over subsequent rounds (10.8% in 2009 and 9.5% in 2010) [2]. With these participation rates, screening was believed to be not (cost-) effective and therefore further nationwide implementation of the program was discontinued [3]. In the present study, we assessed whether the participation rate could be improved by optimizing the invitation letter through systematically applied behavior change theories and evidence [4].

The first step in planned behavior change is to identify the reasons or determinants of the behavior. In an earlier study [5], we assessed the reasons for non-participation by asking 713 people within the age range of 16 and 29 years about their intention to participate in chlamydia screening and included measures of attitude, subjective norm, self-efficacy, the moral norm, susceptibility, the descriptive norm, outcome expectations, and unrealistic optimism toward chlamydia testing. Questions asked were based on literature reports suggesting that the targeted young people felt invulnerable to chlamydia infection, did not compare themselves with people who get infected, and had no time or interest in participating [6,7], experienced barriers such as lack of knowledge, worries, and lack of guidance [8], were afraid of doing it wrong, found the procedure unpleasant, were afraid of the consequences of a possible positive outcome, and expressed fear of negative reactions from a partner and others [9-11]. Furthermore, three single category implicit association tasks (scIAT) [12] were included to identify impulsive reactions towards chlamydia in terms of annoyance, threat, and reassurance. All data were gathered without giving any information in advance about chlamydia or chlamydia testing. The results showed that people have a very low intention to participate in chlamydia screening (mean 1.42, SD 0.76 on a scale of 1-5), low risk perception, in particular low susceptibility, and high unrealistic optimism (most young people do not think they have ever run the risk of being infected with chlamydia and they do not identify themselves with people who test positive for chlamydia). The intention was correlated with the subjective norm, the moral norm, susceptibility, the descriptive norm, one’s attitude, outcome expectations, and unrealistic optimism. Furthermore, chlamydia screening was implicitly associated with reassurance, as well as with threat and annoyance.

Also in the same study, a first attempt was made to optimize the invitation letter by assessing the influence of the original PHS invitation letter versus a letter that was adapted to improve readability and increase a positive response. The results showed no differences between the effects of the two letters; however, receiving a letter had, compared to not receiving a letter, a positive effect on people’s evaluations and intentions to request a test package [5]. There was no measure of testing behavior in that study.

Interventions targeting behavior change have a higher chance of success when theories are systematically applied [13-17]. In the current study, the research question is whether another newly developed invitation letter, systematically written based on theory and adapted to the new evidence from our earlier study has a positive influence on people’s chlamydia screening behavior.

## Methods

### Study Population and Study Procedure

The PHS sent 13,551 letters to all 16 to 29 year old citizens of the Dutch municipality Sittard-Geleen. They received randomly either a newly developed letter or the letter that was already used in the Dutch national Chlamydia Screening Implementation program (CSI). Both invitation letters offered the recipient the opportunity to anonymously request a free chlamydia test kit via a website. At the website, visitors logged in using an anonymous personalized ID and first filled in an 8-item risk questionnaire [18]. Only participants with at least a minimum level of chlamydia risk could proceed to request a test kit. When requested online, they received a chlamydia test, could perform the test at home (urine sample or vaginal swab), send it anonymously to a laboratory, and, within two weeks, they could review their test results online. The study design was approved by the Research Ethics Board of the Faculty of Psychology & Neurosciences of Maastricht University. Registration of this trial was not required.

### Two Invitation Letters

#### Overview

Influencing behavior is more successful when theory is applied and when the content of the message is tailored to the target group [4]. Elaboration Likelihood Theory [19] suggests that people only process information seriously when they are motivated and able to do so. The Reasoned Action Approach [20] suggests that people will change if the right beliefs are changed: beliefs identified through elicitation research. The new letter therefore provides personally relevant information (increase motivation) in such a way that it is easy to process (increase ability) and is tailored to the beliefs that were found in earlier studies (elicitation): risk perception, advantages and disadvantages (attitude), moral norm, social influence, and response- and self-efficacy. There is also anticipation of possible unwanted reactance when people feel pressured and there are prompts to trigger the desired behavior.

The two invitation letters were similar in layout and information content. For the new letter, the order in which information was presented was changed and the content was simplified to increase comprehension and adapted based on the findings of our earlier study [5] and appropriate theories [4]. To keep the new letter short, readers were referred to the website for detailed instructions. Logos on the new letter were identical but fonts were slightly larger to increase readability. Table 1 shows the differences in letters. In this table, the new letter content is chronologically displayed. The PHS letter is not chronologically displayed, but shows how comparable information was given. The (Dutch) invitation letters can be found in Multimedia Appendices 1-4 and [21].

#### First Impression Bias, Primacy Effect, and Self-Affirmation

People’s attitudes or opinions towards specific information are colored by their first impression [22]. Furthermore, the primacy effect suggests that information that is presented first is often remembered best [23]. Therefore, possible negative triggers, as shown in the PHS letter (AIDS, STI), were removed from the top of the letter and added to the footnote in the new letter (see Table 1, #1). Receiving an invitation letter to participate in chlamydia screening can be seen as threatening health information. It is known that people rarely change their behavior after receiving threatening health messages and sometimes the information leads to defensive responses [24]. Self-affirmation is used to decrease the chance of defensive reactions to the threat, or reactance [25]. Applying self-affirmation theory, people were made aware of the value of their personal relationships, thereby increasing their self-identity and promoting a constructive response (see Table 1, #2).

#### Advantages and Convenience, Reactance (I), Efficacy, and the Prompt to Action

Both the advantages (attitude) and ease of testing (self-efficacy) are correlated with the intention to request a chlamydia test package [5]. Therefore, both were highlighted in the newly developed letter (see Table 1, #3). Further, the use of the wording “sexually active” (see Table 1, #2) might be interpreted defensively by receivers as having sex with multiple partners. Moreover, reactance theory suggests that people respond negatively to others’ attempts to limit their freedom [26]. In the new letter, that phrase was deleted and, to prevent possible reactance, the invitation was presented as a general request to all people in that age group, along with a rationale (see Table 1, #4). Also, adaptive behavior is promoted by stressing the belief that the behavior is effective in reducing threat (response efficacy) and the confidence that one can accomplish this behavior (self-efficacy) [24]. Furthermore, a trigger to action was given [27] (see Table 1, #5).

#### Negative Consequences, Severity, and Moral Norm

Threat is the combination of severity (how bad are the consequences?) and susceptibility (do I personally run a risk?) [24]. The severity of the negative consequences of chlamydia is not always recognized [5]; in the letter, the need for early treatment was stressed (see Table 1, #6). Also, in the earlier study, the personal moral norm (a person’s judgment as to whether they themselves think they should or should not perform a certain behavior [28]) was highly correlated with one’s intention to screen. To activate a moral norm, the possibility of unintentionally infecting someone else was mentioned in the letter (see Table 1, #7).

#### Perceived Risk, Unrealistic Optimism, and Reactance (II)

In the earlier study, people scored very low on the perceived risk of getting chlamydia. Furthermore, people thought that other people’s risks were higher than their own. Therefore, it was important to emphasize that all sexually active people, not only people with multiple partners and people who have unsafe sex, can get chlamydia; risk is a matter of risk behavior rather than of risk groups [27] (see Table 1, #8). To minimize a possible reactance (see Reactance (I) above), it was highlighted again that the invitation for a chlamydia test was not a targeted invitation, but part of a regional screening (see Table 1, #9).

#### Self-Efficacy (II) and Procedure, Descriptive Norm, and Implicit Attitudes

In our earlier study, a large majority of people stated that they were unable to test because they did not have time. Therefore, it was important to explain that the procedure would be very simple and would take less than five minutes (see Table 1, #10). Also, people’s behavior, and especially young people’s behavior, is influenced by the behavior of peers [29,30]. Therefore, the letter mentioned that many comparable young people had already tested for chlamydia. Because the earlier study showed an implicit association with annoying, threatening, as well as reassuring, those comparable others were reported to evaluate the test as reassuring and not as threatening or annoying (see Table 1, #11).

#### Moral Norm (II), Anticipated Regret, Privacy, and Response Efficacy

Moral norm, as well as anticipated regret (having people imagine how they would feel after they behaved in a risky way contrary to their own intentions [31]) may both lead to attitude and behavior change (see Table 1, #12). On the topic of privacy, doing a chlamydia test is for many people a private procedure that should not be observable by others [32]. Therefore, the privacy of the testing procedure was stressed in the letter (see Table 1, #13). Also, as mentioned before, a threat may lead to an appropriate behavioral response when people believe that such a response is available and easy to do (see Table 1, #14).

#### Log-In Code, Prompt to Action, Sender Information, and Footnote Information

The information about the log-in procedure was simplified in the new letter (see Table 1, #15). Also included were prompts to form a plan for action, which may increase the number of people performing the testing behavior [33] (see Table 1, #16). Sender information was identical in both letters (see Table 1, #17), but footnote information was simplified and, to avoid primacy effects, the AIDS/STD info was given here (see First Impression Bias and Primacy Effect above and Table 1, #18).

**Table 1:** Adaptation of the new letter compared to the original PHS letter.

New letter		Comparable information from PHS letter
**1. First Impression Bias and Primacy Effects**		
	Visiting address: Het Overloon 2 6411 TE Heerlen 045-8506613 (9.00h-12.00h)	Visiting Address: Het Overloon 2 6411 TE Heerlen 045-850 66 13 (9.00h-12.00h) Aids STD Info line (for questions about sex, STD and The Pill): 0900-2042040
**2. Self-Affirmation**		
	“Your health is very important. Not only for yourself, but also for a possible partner and family.”	“When you are (or have been) sexually active, it is important to do this test, even if you’ve done this test before or if you’re in a solid relationship.”
**3. Advantages and Convenience**		
	“Therefore, you should take the opportunity to take a free and easy chlamydia test at home.”	“Via this letter, we invite you again to participate in the Chlamydia Screening South-Limburg.”
**4. Reactance (I)**		
	“When you have sex, it is possible to contract chlamydia without realizing it.”	“That [ie, treat quickly] is difficult, because most people don’t know whether they are infected with chlamydia or not.”
**5. Efficacy and Prompt to Action**		
	“Chlamydia is simple and easy to trace, and very trouble-free to treat. However, it is important to do the test as early as possible!”	“It is easy to treat, but important to be quick. Severe and frustrating health issues can be prevented by testing for chlamydia.”
**6. Negative Consequences, Severity**		
	“If you wait too long for treatment, you can get severe and permanent health issues like infertility.”	“If chlamydia is not treated in time, men can get epididymitis and women can become infertile.”
**7. Moral Norm**		
	“Above all, you can infect others without knowing it.”	“That [ie, treat quickly] is difficult, because most people don’t know whether they are infected with chlamydia or not.”
**8. Perceived Risk, Unrealistic Optimism**		
	“Chlamydia does not only occur in people who have unsafe sex with many partners, but also in people with a few partners or just one partner.”	“Chlamydia is an STD (sexually transmitted disease) with a high prevalence in the Netherlands, especially in young people aged 16 to 29.”
**9. Reactance (II)**		
	“To decrease the number of chlamydia infections, all 16-29 year olds from your region are invited to request a free chlamydia test via www.chlamydiatest.nl.”	“On www.chlamydiatest.nl The PHS South Limburg invites all men and women, aged 16-29 years, to participate in the annual free chlamydia test. On www.chlamydiatest.nl, ”
**10. Self-efficacy (II) and Procedure**		
	“When you have requested and received the test package, you can do the test in less than five minutes at home, after which you can send it to the laboratory.”	“How does it work: You log in on with your personal log-in code from this letter. There you can create immediately your own username and password, making sure that no one else can log in. Subsequently, you can request a test package. You will receive this in a blank package at the address of your choice. You will find instructions in the test package about how to collect your test sample. Subsequently, you can send the package back to the laboratory at no cost. The test result is available within two weeks and will be online for three months via www.chlamydiatest.nl. With your username and password, you can request your test result. If you’ve forgotten your personal details, you need your personal log-in code from this letter. Therefore, you should keep this letter!”
**11. Descriptive Norm and Implicit Attitudes**		
	“A lot of your peers have already tested for chlamydia, not finding it annoying or threatening, but more reassuring.”	N/A
**12. Moral Norm (II) and Anticipated Regret**		
	“Also, some of them said that they would feel guilty if they didn’t do the test.”	N/A
**13. Privacy**		
	“The entire test can be performed anonymously and at no charge. Your personal details are kept confidential. Only you, with your username and password, are able to request your test result (within two weeks!). No one, not your parents nor your general practitioner, is notified of the results.”	“All your personal details are kept confidential. Only you, with your username and password, are able to request your test result. And only you, not your parents nor your general practitioner, are notified of the results. In the folder, attached to this letter, and on www.chlamydiatest.nl, you can find more information about chlamydia and the test.”
**14. Response Efficacy**		
	“If you are infected with chlamydia, it is very easily treated with antibiotics.”	N/A
**15. Log-in code**		
	“Your strictly personal log-in code for www.chlamydiatest.nl is: ”	“To log in, you need a personal log-in code. Your strictly personal log-in code for www.chlamydiatest.nl is: ”
**16. Prompt to Action (II)**		
	“Don’t delay − request your test package today!”	“Think about your health and participate in the chlamydia screening!”
**17. Sender Information**		
	“With kind regards, Dr. Christian J.P.A. Hoebe Doctor/Epidemiologist, Infectious Diseases Project Leader, Chlamydia Screening South Limburg”	“With kind regards, Dr. Christian J.P.A. Hoebe Doctor/Epidemiologist, Infectious Diseases Project Leader, Chlamydia Screening South Limburg”
**18. Footnote Information**		
	“For questions and information: www.chlamydiatest.nl. For questions about participation: ms H.L.G. ter Waarbeek (independent GP: 045-8506264). For questions about sex, STDs and The Pill: Aids STD info line: 0900-2042040.”	“There (ie, on www.chlamydiatest.nl can read about all the things you have to do for this test. P.S. This invitation is sent district by district across 10 municipalities to all individuals aged 16 to 29 years, so not everybody receives the invitation at the same time. If you want to know which municipalities, see www.chlamydiatest.nl. If you want to discuss questions about participation in this study with an independent general practitioner: contact ms H.L.G. ter Waarbeek (045-8506264).”

## Results

**Figure 1:**
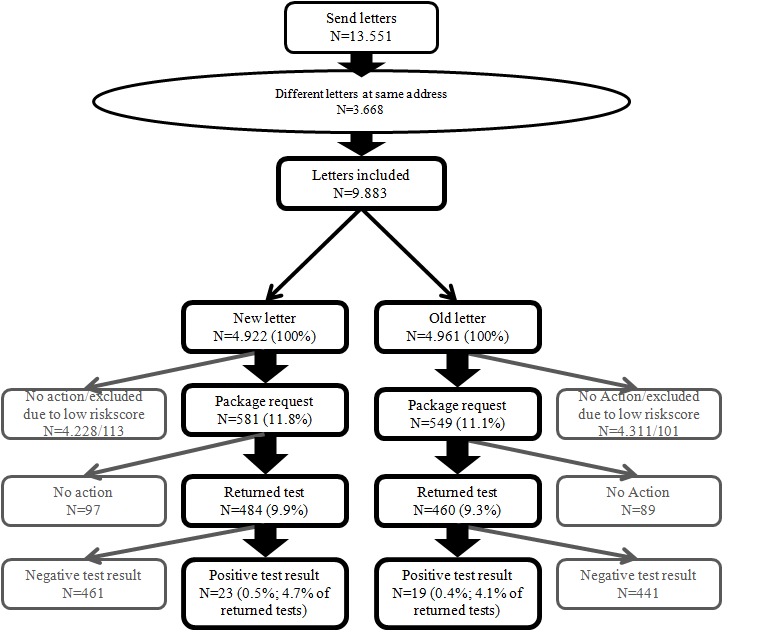
Flowchart of responses to the new letter and the original PHS letter.

In total, 13,551 letters were sent to all 16 to 29 year old citizens of the municipality Sittard-Geleen (population 94,024 [34]) in the south of the Netherlands, randomly divided over the new and the original letter. When two different letters were delivered at one unique address (which could only be checked afterward), or when letters were returned as undeliverable, these data were excluded from further analyses (n=3668). Of the 9883 included respondents, 11.43% (1130/9883) requested a test package. No significant differences in test package requests were found between the two letters (χ^2^_1_=1.33, *P*=.25, phi= −.012). There were also no differences between the two letters for the rates of returned tests (χ^2^_1_=0.05, *P*=.82, phi= .007), and the number of positive test results (χ^2^_1_=0.21, *P*=.64, phi= −.015) (see Figure 1). It is evident that the new letter did not improve participation compared to the original letter. In acknowledgement of recent concerns regarding lack of disclosure in scientific research [35], and to aid future meta-analyses, all data, syntax files, and output files are available in Multimedia Appendices 1- 6 and [21].

## Discussion

### Principal Findings

In this study, the effect of a new theory- and evidence-based adaptation of an invitation letter for chlamydia screening was compared to the effect of the original letter. Both letters resulted in small percentages of participation, comparable to other screening projects in the Netherlands [3] and outside the Netherlands [36]. However, contrary to expectations, there was no significant difference between the two letters. The new letter did not stimulate more young people to go for the test.

### Strengths and Limitations

The strength of this study is that actual behavior was measured, while the weakness is that there was only observational data and no data on how people processed the information or on possible changes in the determinants of behavior. It is, however, difficult to imagine how a mass media letter could be improved differently to reach a substantial larger number of young people participating in the screening. It is obvious that the approach to invite the target population via a general letter does not lead to sufficient participation rates for chlamydia screening.

### Conclusions

Why is it so hard to convince young people to participate in chlamydia screening? The strongest determinants of chlamydia screening participation in earlier studies seem to be (low) risk perception, in particular low susceptibility, and high unrealistic optimism [5]. That means that the basic proposition for action is fully lacking [24] and, as it turns out, it seems to be very difficult to convince people that they indeed are at risk. Risk perception and unrealistic optimism can be changed but not easily. Bartholomew et al [4] suggest a number of methods including scenario-based risk information, consciousness raising, or self-affirmation, but those methods require more individual tailoring, more attention, and more time than is feasible in one general letter (page 333 [4]). Other behavior change approaches may be needed. There are some suggestions in the literature: the use of the Internet independent of geographic area [37], financial incentives [38], a focus on self-identity [39], and tailoring on risk perception [40]. Schmid et al [3] suggest retesting people who were found positive and intensifying partner notification. In that approach, the focus is on people who already know they are at risk. Based on the results in our studies, possible alternative strategies for people who do not see themselves at risk might involve the use of social media in targeting high-risk groups. Social circles around people who test positive for chlamydia are shown to be at higher risk [41,42]. Young people who tested positive in chlamydia screening could serve as role models for other young people in their social circles. If this approach is used, the target group should see those models as someone from their own circle that they can identify with, who had to overcome some personal resistance to participate, who is reinforced for participating in the screening by reporting reassurance, and who explains the ease of participation [43]. This alternative approach should be tried out in a randomized study comparable to this study.

## Multimedia Appendix 1

New letter.

## Multimedia Appendix 2

New letter translated.

## Multimedia Appendix 3

PHS letter.

## Multimedia Appendix 4

PHS letter translated.

## Multimedia Appendix 5

Datafile.

## Multimedia Appendix 6

SPSS syntax.

## Abbreviations

CSI: Chlamydia Screening Implementation program
PHS: public health services
STD: sexually transmitted disease

## References

[1] Chlamydia Screening Website.

[2] van den Broek IV, van Bergen JE, Brouwers EE, Fennema JS, Götz HM, Hoebe CJ, Koekenbier RH, Kretzschmar M, Over EA, Schmid BV, Pars LL, van Ravesteijn SM, van der Sande MA, de Wit GA, Low N, Op de Coul EL (2012). Effectiveness of yearly, register based screening for chlamydia in the Netherlands: controlled trial with randomised stepped wedge implementation. BMJ.

[3] Schmid BV, Over EAB, van den Broek IVF, Op de Coul ELM, van Bergen JEAM, Fennema JSA, Götz HM, Hoebe CJPA, de Wit GA, van der Sande MAB, Kretzschmar MEE (2013). Effects of population based screening for Chlamydia infections in the Netherlands limited by declining participation rates. PLoS One.

[4] Bartholomew LK, Parcel GS, Kok G, Gottlieb NH, Fernández ME (2011). Planning Health Promotion Programs: An Intervention Mapping Approach, 3rd Edition.

[5] Ten Hoor GA, Ruiter RA, van Bergen JE, Hoebe CJ, Houben K, Kok G (2013). Non-participation in chlamydia screening in the Netherlands: determinants associated with young people's intention to participate in chlamydia screening. BMC Public Health.

[6] Duncan B, Hart G, Scoular A, Bigrigg A (2001). Qualitative analysis of psychosocial impact of diagnosis of Chlamydia trachomatis: implications for screening. BMJ.

[7] Greenland KE, Op de Coul EL, van Bergen JE, Brouwers EE, Fennema HJ, Götz HM, Hoebe CJ, Koekenbier RH, Pars LL, van Ravesteijn SM, van den Broek IV (2011). Acceptability of the internet-based Chlamydia screening implementation in the Netherlands and insights into nonresponse. Sex Transm Dis.

[8] McNulty CA, Freeman E, Bowen J, Shefras J, Fenton KA (2004). Barriers to opportunistic chlamydia testing in primary care. Br J Gen Pract.

[9] PHS, Public Health Service Amsterdam (2010) (2012). Kwalitatief onderzoek naar redenen voor het niet terugsturen van een aangevraagd Chlamydia testpakket; Chlamydia Screening Implementation (CSI) A qualitative study on reasons for not returning a requested chlamydia screening test. Public Health Service Amsterdam (2010) (study report).

[10] Blake DR, Kearney MH, Oakes JM, Druker SK, Bibace R (2003). Improving participation in Chlamydia screening programs: perspectives of high-risk youth. Arch Pediatr Adolesc Med.

[11] Rose SB, Smith MC, Lawton BA (2008). "If everyone does it, it's not a big deal." Young people talk about chlamydia testing. N Z Med J.

[12] Karpinski A, Steinman RB (2006). The single category implicit association test as a measure of implicit social cognition. J Pers Soc Psychol.

[13] Albarracín D, Gillette JC, Earl AN, Glasman LR, Durantini MR, Ho MH (2005). A test of major assumptions about behavior change: a comprehensive look at the effects of passive and active HIV-prevention interventions since the beginning of the epidemic. Psychol Bull.

[14] de Bruin M, Viechtbauer W, Schaalma HP, Kok G, Abraham C, Hospers HJ (2010). Standard care impact on effects of highly active antiretroviral therapy adherence interventions: A meta-analysis of randomized controlled trials. Arch Intern Med.

[15] Mullen PD, Green LW, Persinger GS (1985). Clinical trials of patient education for chronic conditions: a comparative meta-analysis of intervention types. Prev Med.

[16] Peters LW, Kok G, Ten Dam GT, Buijs GJ, Paulussen TG (2009). Effective elements of school health promotion across behavioral domains: a systematic review of reviews. BMC Public Health.

[17] van Achterberg T, Huisman-de Waal GG, Ketelaar NA, Oostendorp RA, Jacobs JE, Wollersheim HC (2011). How to promote healthy behaviours in patients? An overview of evidence for behaviour change techniques. Health Promot Int.

[18] van den Broek IV, Brouwers EE, Götz HM, van Bergen JE, Op de Coul EL, Fennema JS, Koekenbier RH, Pars LL, van Ravesteijn SM, Hoebe CJ (2012). Systematic selection of screening participants by risk score in a Chlamydia screening programme is feasible and effective. Sex Transm Infect.

[19] Petty RE, Barden J, Wheeler SC, DiClemente RJ, Crosby RA, Kegler M (2009). The Elaboration Likelihood Model of Persuasion: Developing health promotions for sustained behavioral change. Emerging Theories in Health Promotion Practice and Research.

[20] Fishbein M, Ajzen I (2010). Predicting and changing behavior: the reasoned action approach.

[21] ScienceRep Repository (Multimedia Appendices).

[22] Lim KH, Benbasat I, Ward LM (2000). The role of multimedia in changing first impression bias. Information Systems Research.

[23] Petty RE, Wegener DT, Gilbert DR, Fiske ST, Lindzey G (1998). Attitude change: multiple roles for persuasion variables. The handbook of social psychology.

[24] Peters GJ, Ruiter RA, Kok G (2013). Threatening communication: a critical re-analysis and a revised meta-analytic test of fear appeal theory. Health Psychol Rev.

[25] Harris PR, Mayle K, Mabbott L, Napper L (2007). Self-affirmation reduces smokers' defensiveness to graphic on-pack cigarette warning labels. Health Psychol.

[26] Laurin K, Kay AC, Fitzsimons GJ (2012). Reactance versus rationalization: divergent responses to policies that constrain freedom. Psychol Sci.

[27] Champion VL, Skinner CS, Glanz K, Rimer BK, Viswanath K (2008). The health belief model. Health behavior and health education: theory, research, and practice.

[28] Godin G, Fortin C, Michaud F, Bradet R, Kok G (1997). Use of condoms: Intention and behaviour of adolescents living in juvenile rehabilitation centres. Health Education Research.

[29] Cialdini RB (2009). Influence: science and practice.

[30] Mollen S, Rimal RN, Ruiter RA, Jang SA, Kok G (2013). Intervening or interfering? The influence of injunctive and descriptive norms on intervention behaviours in alcohol consumption contexts. Psychol Health.

[31] Richard R, van der Pligt J, de Vries N (1995). Anticipated affective reactions and prevention of AIDS. Br J Soc Psychol.

[32] Westmaas AH, Kok G, Vriens P, Götz H, Richardus JH, Voeten H (2012). Determinants of intention to get tested for STI/HIV among the Surinamese and Antilleans in the Netherlands: results of an online survey. BMC Public Health.

[33] Milkman KL, Beshears J, Choi JJ, Laibson D, Madrian BC (2012). Following through on good intentions: The power of planning prompts. National Bureau of Economic Research, MA: Cambridge.

[34] Deerenberg I, Elzinga H, van Houwelingen C, Keuning - van Oirschot H, Geurden - Slis M, Beeckman D, Raets B, Apperloo J (2011). Centraal Bureau voor de statistiek.

[35] Peters GJY, Abraham C, Crutzen R (2012). Full disclosure: doing behavioural science necessitates sharing. European Health Psychologist.

[36] Jamil MS, Hocking JS, Bauer HM, Ali H, Wand H, Smith K, Walker J, Donovan B, Kaldor JM, Guy RJ (2013). Home-based chlamydia and gonorrhoea screening: a systematic review of strategies and outcomes. BMC Public Health.

[37] Woodhall SC, Sile B, Talebi A, Nardone A, Baraitser P (2012). Internet testing for Chlamydia trachomatis in England, 2006 to 2010. BMC Public Health.

[38] Currie MJ, Schmidt M, Davis BK, Baynes AM, O'Keefe EJ, Bavinton TP, McNiven M, Martin SJ, Bowden FJ (2010). 'Show me the money': financial incentives increase chlamydia screening rates among tertiary students: a pilot study. Sex Health.

[39] Booth AR, Norman P, Harris PR, Goyder E (2013). Using the theory of planned behaviour and self-identity to explain chlamydia testing intentions in young people living in deprived areas. Br J Health Psychol.

[40] Mevissen FE, Ruiter RA, Meertens RM, Zimbile F, Schaalma HP (2011). Justify your love: testing an online STI-risk communication intervention designed to promote condom use and STI-testing. Psychol Health.

[41] Rosenberg NE, Pettifor A, Kamanga G, Bonongwe N, Mapanje C, Hoffman I, Martinson F, MIller WC (2013). Social networks of STI patients have higher STI prevalence than social networks of community controls. Sexually Transmitted Infections.

[42] Theunissen K, Hoebe C, Crutzen R, Niekamp A, Kara-Zaitri C, Vries ND, Bergen JV, Sande MBVD, Dukers-Muijrers N (2013). A targeted Web-based Chlamydia Trachomatis screening strategy for testing in young people at risk using social and sexual networks. Sexually Transmitted Infections.

[43] McAlister AL, Perry CL, Parcel GS, Glanz K, Rimer BK, Viswanath K (2008). How individuals, environments,health behaviors interact: Social Cognitive Theory. Health behavior and health education: theory, research, and practice (4th edition).

