# Supplementary figures and images for: The Influence of Two Different Invitation Letters on Chlamydia Testing Participation: Randomized Controlled Trial
Source: J Med Internet Res. 2014 Jan 30;16(1):e24. doi: 10.2196/jmir.2907 (PMC3936267; doi:10.2196/jmir.2907)

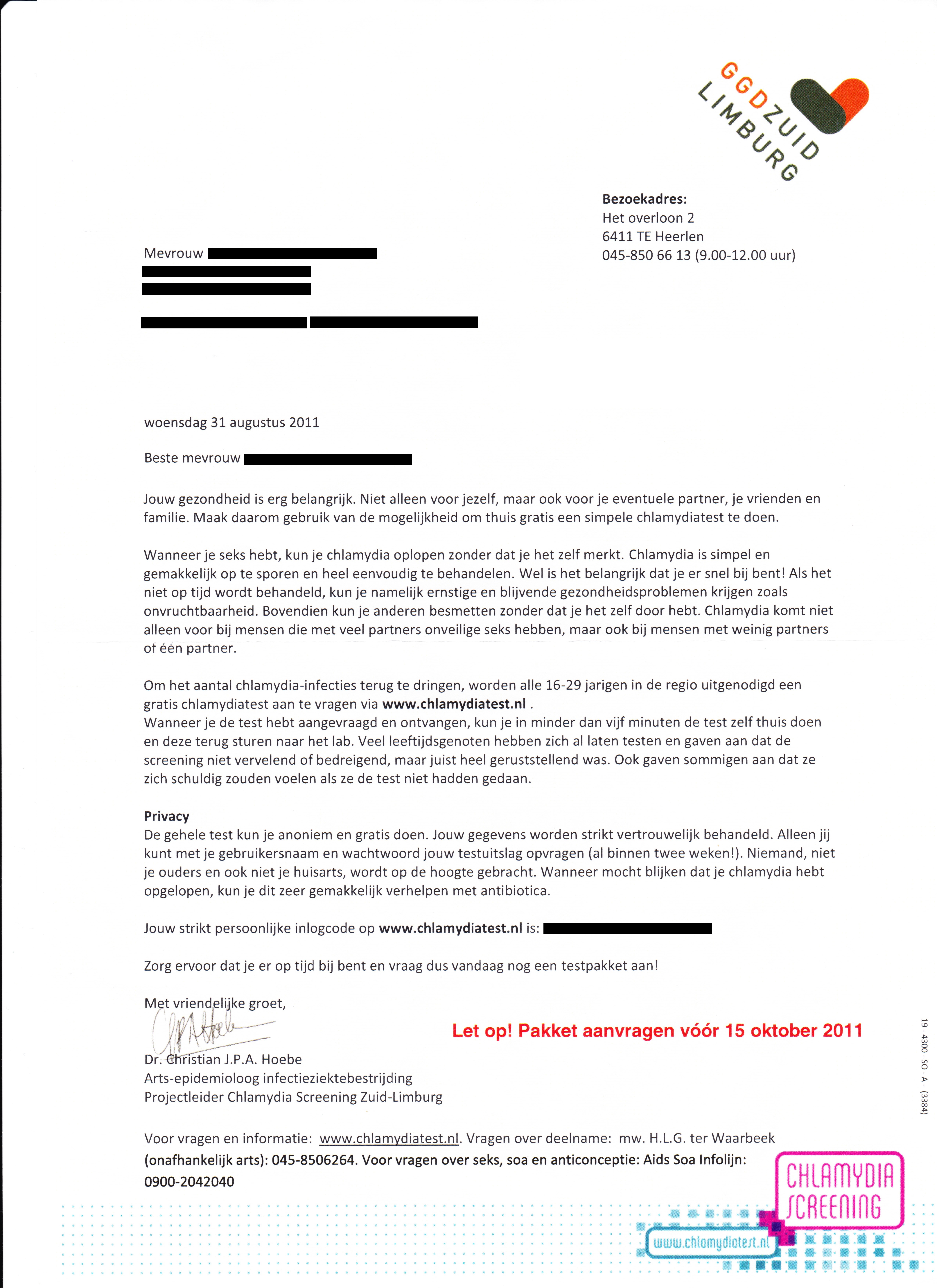

Supplement: Supplementary file 1 [file jmir_v16i1e24_app1.jpg]
