# Supplementary material for: The Influence of Two Different Invitation Letters on Chlamydia Testing Participation: Randomized Controlled Trial
Source: J Med Internet Res. 2014 Jan 30;16(1):e24. doi: 10.2196/jmir.2907 (PMC3936267; doi:10.2196/jmir.2907)
Supplement: Supplementary file 2 [file jmir_v16i1e24_app2.pdf]

Visiting address:
Het Overloon 2
6411 TE Heerlen
045-8506613 (9.00h-12.00h)

Dear ….

Your health is very important. Not only for yourself, but also for a possible partner, and your family. Therefore, you should take the opportunity to do a chlamydia test at home. It's free of charge and easy to do.

If you have sex, it is possible to contract chlamydia without noticing it. Chlamydia is simple and easy to trace, and very easy to treat. However, it is important to do the test as early as possible! If you wait too long for treatment, you can get severe and permanent health issues such as infertility. Above all, you can infect others, without knowing it. Chlamydia does not only infect people who have unsafe sex with many partners, but it also infects people with a few partners or just one partner.

To reduce the amount of chlamydia infections, all 16-29 year olds from your region have been invited to request a free chlamydia test via www.chlamydiatest.nl.
When you have requested and received a test package, you can do the test in less than five minutes at home after which you can send the test back to the laboratory. A lot of your peers have already tested for chlamydia, and they did not experience it as annoying or threatening, but as reassuring. Also, some of them said that they would feel guilty if they did not do the test.

The test can be performed anonymously and free of charge. Your personal details are processed confidentially. Only you, with your username and password, are able to request your test result (already within two weeks!). Not anybody, including you parent or your general practitioner is informed of your test results. Chlamydia is very easy to treat with antibiotics.

Your strictly personal login code for www.chlamydiatest.nl is: xxxxx
Make sure to be on time and request your test package today!

With kind regards,

Dr. Christian J.P.A. Hoebe
Doctor-epidemiologist infectious diseases.
Project leader Chlamydia Screening South Limburg

For questions and information: www.chlamydiatest.nl. For questions about participation: ms H.L.G. ter Waarbeek (independent GP: 045-8506264). For questions about sex, STD's and The Pill: Aids STD info line: 0900-2042040.
