# Supplementary material for: The Influence of Two Different Invitation Letters on Chlamydia Testing Participation: Randomized Controlled Trial
Source: J Med Internet Res. 2014 Jan 30;16(1):e24. doi: 10.2196/jmir.2907 (PMC3936267; doi:10.2196/jmir.2907)
Supplement: Supplementary file 3 [file jmir_v16i1e24_app3.pdf]

GGD ZUID LIMBURG

Bezoekadres:
Het overloon 2
6411 TE Heerlen
045-8506613 (9.00-12.00 uur)
Aids Soa Infolijn: (voor vragen over seks, soa en de pil):
0900-2042040

Datum:

Beste

Via deze brief nodigen we je uit deel te nemen aan de Chlamydia Screening Zuid-Limburg.
Chlamydia is een soa (seksueel overdraagbare aandoening) die in Nederland veel voorkomt, met name bij jonge mensen in de leeftijd van 16 tot en met 29 jaar. Chlamydia is eenvoudig te behandelen, maar het is belangrijk om er snel bij te zijn. Dat is lastig, want de meeste mensen merken niet dat zij chlamydia hebben. Als chlamydia niet op tijd wordt behandeld, kun je vervelende en ernstige gezondheidsklachten krijgen (bijvoorbeeld bijbalontsteking voor mannen en onvruchtbaarheid voor vrouwen).

De GGD Zuid Limburg wil het aantal chlamydia-infecties terugdringen. Daarom worden alle mannen en vrouwen van 16 tot en met 29 jaar in Zuid-Limburg uitgenodigd om een eenvoudige, gratis chlamydiatest te doen. Deze brief is jouw uitnodiging.
Als je seksueel actief bent (geweest), is het belangrijk om je te laten testen, ook als je op dit moment een vaste relatie hebt .
Om mee te doen, heb je een testpakket nodig. Dat kun je heel gemakkelijk aanvragen via de website www.chlamydiatest.nl.
Gebruik hiervoor onderstaande persoonlijke inlogcode.

Jouw strikt persoonlijke inlogcode op www.chlamydiatest.nl is:

Hoe werkt het?
Als je een testpakket hebt aangevraagd, krijg je deze in een blanco verpakking opgestuurd naar het door jou opgegeven adres. In het testpakket vind je instructies hoe je zelf testmateriaal kunt verzamelen. Vervolgens stuur je het pakketje gratis voor onderzoek naar het laboratorium.
De uitslag is binnen twee weken bekend en kun je bekijken op www.chlamydiatest.nl. Je hebt je gebruikersnaam en wachtwoord nodig om op de site in te loggen. Deze maak je na de 1e keer inloggen zelf aan. Als je je gegevens kwijtraakt heb je je persoonlijke inlogcode uit deze brief nodig. **Bewaar deze brief daarom goed!**

Privacy
Al je gegevens worden strikt vertrouwelijk behandeld. Alleen jij kunt met je gebruikersnaam en wachtwoord jouw testuitslag opvragen. Niemand, niet je ouders en ook niet je huisarts, wordt op de hoogte gebracht. In de folder bij deze brief en op www.chlamydiatest.nl vind je meer informatie over chlamydia en de test. Denk aan je gezondheid en doe mee aan de Chlamydia Screening Zuid-Limburg!

Met vriendelijke groet,
dr. Christian J.P.A. Hoebe
Arts-epidemioloog infectieziektebestrijding

Projectleider Chlamydia Screening Zuid-Limburg

Ps: Deze uitnodiging wordt in 10 gemeentes wijk voor wijk verstuurd naar alle mannen en vrouwen van 16 tot en met 29 jaar, dus niet iedereen ontvangt op hetzelfde moment een uitnodiging. Als je wilt weten om welke gemeentes het gaat, kijk dan op www.chlamydiatest.nl.
Mocht je vragen over deelname aan dit onderzoek willen bespreken met een onafhankelijk arts, neem contact op mw. H.L.G. ter Waarbeek: 045-8506276

CHLAMYDIA SCREENING
www.chlamydiatest.nl
