# Supplementary material for: The Influence of Two Different Invitation Letters on Chlamydia Testing Participation: Randomized Controlled Trial
Source: J Med Internet Res. 2014 Jan 30;16(1):e24. doi: 10.2196/jmir.2907 (PMC3936267; doi:10.2196/jmir.2907)
Supplement: Supplementary file 4 [file jmir_v16i1e24_app4.pdf]

Visiting Address:
Het Overloon 2
6411 TE Heerlen
045-850 66 13 (9.00h-12.00h)
Aids STD Info line (for questions about sex, STD and The Pill):
0900-2042040

Dear…

Via this letter, we invite you to participate in the Chlamydia Screening South-Limburg. Chlamydia is a STD (Sexually Transmitted Disease) with a high prevalence in the Netherlands, especially in young people aged 16 to 29 years old. It is easy to treat, but it is important to receive treatment quickly. That is difficult, because most people don't know whether they are infected with chlamydia or not. If chlamydia is not treated in time, men can get epididymitis and women can become infertile. Severe and frustrating health issues can be prevented by testing for chlamydia.

The PHS South Limburg wants to reduce the amount of Chlamydia infections. Therefore, all men and women aged 16-29 years old in South Limburg are invited to do a simple, free chlamydia test. This letter is your invitation. When you are (or have been) sexually active, it is important to do this test, even when you've done this test earlier, or when you're having a steady relationship.
To participate you need a test package. On www.chlamydiatest.nl, you can very easily request a test package. For this, you need to use your personal login code.

Your strictly personal login code for www.chlamydiatest.nl is: xxxxx

How does it work?
You log in on www.chlamydiatest.nl with your personal login code from this letter. There you can immediately create your own username and password, making sure that no one else can log in. Subsequently, you can request a test package. You will receive this in a blank package at the address of your choice. In the test package, you will find instructions about how to collect your testing material. After this, you can send the package back to the laboratory without any costs. The test result is available within two weeks and will be online for three months via www.chlamydiatest.nl. With your username and password, you can request your test result. When you've forgotten your personal details, you need your personal login code from this letter. **Therefore, you should keep this letter in a safe place!**

Privacy
All your personal details are kept confidential. Only you, with your username and password, are able to request your test result. And only you, not your parents or your general practitioner is informed of your test results.
In the folder attached to this letter, and on www.chlamydiatest.nl, you can find more information about chlamydia and the test.

Think about your health and participate in the chlamydia screening!

With kind regards,

Dr. Christian J.P.A. Hoebe
Doctor-epidemiologist infectious diseases.
Project leader Chlamydia Screening South Limburg

P.S. this invitation is send - district after district - to ten municipalities to all youngsters aged 16 to 29 year old, so not everyone receives the invitation at the same time. If you want to know which municipalities those are, see www.chlamydiatest.nl. If you want to discuss questions about participation in this study with an independent general practitioner: contact ms H.L.G. ter Waarbeek (045-8506264).
